# Research advances on neurite outgrowth inhibitor B receptor

**DOI:** 10.1111/jcmm.15391

**Published:** 2020-06-15

**Authors:** Rui Zhang, Bei‐sha Tang, Ji‐feng Guo

**Affiliations:** ^1^ Department of Neurology Xiangya Hospital Central South University Changsha China; ^2^ Center for Medical Genetics School of Life Sciences Central South University Changsha China; ^3^ National Clinical Research Center for Geriatric Disorders Central South University Changsha China; ^4^ Key Laboratory of Hunan Province in Neurodegenerative Disorders Central South University Changsha China; ^5^ Parkinson's Disease Center Beijing Institute for Brain Disorders Beijing China

**Keywords:** angiogenesis, cholesterol trafficking, NgBR, NUS1, Parkinson's disease, tumorigenesis

## Abstract

Neurite outgrowth inhibitor‐B (Nogo‐B) is a membrane protein which is extensively expressed in multiple organs, especially in endothelial cells and vascular smooth muscle cells of blood vessels and belongs to the reticulon protein family. Notably, its specific receptor, Nogo‐B receptor (NgBR), encoded by *NUS1*, has been implicated in many crucial cellular processes, such as cholesterol trafficking, lipid metabolism, dolichol synthesis, protein N‐glycosylation, vascular remodelling, angiogenesis, tumorigenesis and neurodevelopment. In recent years, accumulating studies have demonstrated the statistically significant changes of NgBR expression levels in human diseases, including Niemann‐Pick type C disease, fatty liver, congenital disorders of glycosylation, persistent pulmonary hypertension of the newborn, invasive ductal breast carcinoma, malignant melanoma, non‐small cell lung carcinoma, paediatric epilepsy and Parkinson's disease. Besides, both the in vitro and in vivo studies have shown that NgBR overexpression or knockdown contribute to the alteration of various pathophysiological processes. Thus, there is a broad development potential in therapeutic strategies by modifying the expression levels of NgBR.

## 1 INTRODUCTION

Nogo‐B was identified as a protein which highly expressed in caveolin‐1 enriched micro‐domains of endothelial cells (EC) and was considered as a family member of reticulons (RTN) that largely restricted to the tubular endoplasmic reticulum (ER) both in yeast and mammalian cells.^1^, ^2^, ^3^ Additionally, Nogo‐B is found in multiple organs, especially in EC and vascular smooth muscle cells (VSMC) of blood vessels.^3^, ^4^

In 2006, Miao and his colleagues identified a previously uncharacterized Nogo‐B receptor (NgBR), encoding by *NUS1* gene in human, specifically binds to the amino terminus of Nogo‐B (AmNogo‐B). Importantly, the binding of Nogo‐B and NgBR involves in stimulating chemotaxis and 3D tube formation and angiogenesis.^5^ Since then, a considerable number of researches have been performed to disentangle the functions of NgBR. To our surprise, NgBR is involved in many other pathophysiological processes such as cholesterol trafficking,^6^ dolichol synthesis,^7^ protein N‐glycosylation,^7^ tumorigenesis^8^, ^9^, ^10^, ^11^ and neurodevelopment,^12^ and other new features of NgBR are constantly being disclosed.

In this review, we will summarize kinds of published literatures from the functional aspects of NgBR to provide a comprehensive understanding of the existing functions of NgBR and, meanwhile, offer possible directions for the next research.

## 2 NgBR, A SPECIFIC RECEPTOR FOR Nogo‐B

**FIGURE 1:**
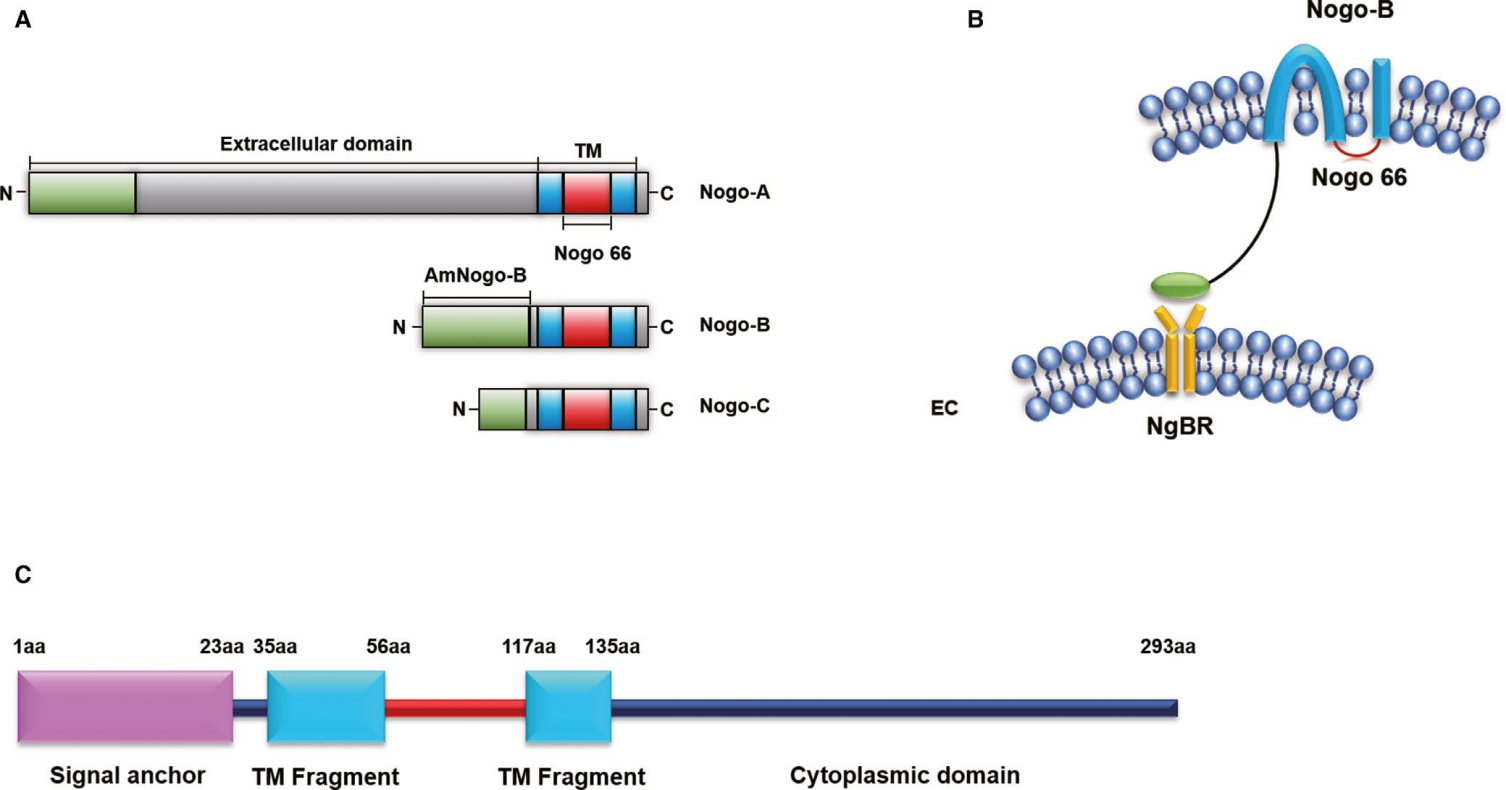
A, Structures of Nogo‐A, Nogo‐B and Nogo‐C. B. NgBR specifically binds to AmNogo‐B on vascular endothelial cells. C, The NgBR domains are identified by Harrison et al.^7^ Endothelial cell (EC)

Previous studies have demonstrated that Nogo‐A, Nogo‐B, and Nogo‐C are three isoforms of RTN4/Nogo protein family. Nogo‐A plays a pivotal role in axonal plasticity and serves as an inhibitor of axonal growth and repair.^13^ By contrast, Nogo‐C is the shortest one among the three Nogo isoforms and it negatively regulates cell proliferation, apoptosis, axonal re‐extension and cardiac functions.^14^, ^15^, ^16^, ^17^ To note, Nogo‐B shares the same Nogo‐66 region with Nogo‐A and Nogo‐C.^18^ It is mainly expressed in EC and VSMC whose function is regulating cell migration and vascular remodelling^3^ (Figure 1A). In addition, soluble Nogo‐B (sNogo‐B) is a circulating isoform of full‐length Nogo‐B, and the overexpression of sNogo‐B could protect the damaged vasculature by vascular remodelling following injury.^19^

Nogo‐B receptor was identified specifically binding to the AmNogo‐B which is a transmembrane protein mainly residing in ER containing 293 residues (Figure 1B,C). Its cytoplasmic domain shows a high similarity to cis‐prenyltransferase (cis‐PTase), a lipid‐modifying enzyme. To further explore the structural properties of NgBR, Li and Song found its ectodomain is intrinsically unstructured. As reported before, the very unusual properties of the intrinsically unstructured family of proteins have been considered to play roles in special categories of biological functions including tumours, signal pathway regulation, protein‐DNA recognition, endocytosis and generating/maintaining membrane structure as well.^20^ Therefore, how does the intrinsically unstructured NgBR ectodomain functions worth to further research. But, its unstructured domain may become well‐structured when binding to Nogo‐B or other partners.^20^ Recent studies have demonstrated that NgBR co‐localizes with Nogo‐B to promote vascular remodelling and angiogenesis both in physiological and pathological conditions.^5^, ^19^ In physiological condition, structure‐function analysis demonstrates NgBR specifically binds to AmNogo‐B, and Nogo‐B/NgBR axis is necessary to regulate vascular remodelling and angiogenesis.^5^ Similarly, in pathological condition such as diabetic kidney disease, sNogo‐B plays an important role in vascular remodelling and angiogenesis when binds to NgBR.^19^ Collectively, the discovery of NgBR as a specific receptor for Nogo‐B has greatly advanced our understanding of the biological functions of the Nogo protein family, and the structural characterization of NgBR will provide more valuable insights in understanding the underlying molecular mechanisms.

## 3 NgBR REGULATES CHOLESTEROL TRAFFICKING AND LIPID METABOLISM

In mammalian cells, cholesterol homeostasis is critical for the maintenance of numerous cellular functions. The internalized cholesterol is then trafficked from endosome to many other cellular structures including lysosomes, ER and other organelles membranes, where it exerts diverse physiological functions.^21^, ^22^

Niemann‐Pick type C disease (NPC) is a lysosomal storage disease with abnormal intracellular cholesterol accumulation and imbalanced sterol homeostasis and finally leads to liver failure and neurodegeneration.^23^ Niemann‐Pick type C1 (NPC1) protein is an intracellular cholesterol transporter that resides primarily in endocytic organelles.^24^ Additionally, Niemann‐Pick type C2 (NPC2) protein is a small soluble glycoprotein that binds to LDL‐released cholesterol and transfers it onto NPC1 and thus plays a critical role in cholesterol trafficking between membranes.^25^, ^26^ Mutations in both NPC1 or NPC2 protein can lead to NPC phenotype.

Excitingly, Harrison et al found that the C‐terminus of NgBR can specifically bind to NPC2. Additionally, further studies showed the same distribution of NPC2 with C‐terminus of NgBR. Moreover, in the presence of NgBR, the half‐life of NPC2 was prolonged from 3.5 to 5.1 hours, indicating increased stability of NPC2. Besides, it was also found that NgBR also functions to stabilize nascent NPC2. Finally, knockdown of NgBR resulted in increasing free cholesterol but reducing NPC2 levels, which can be rescued by transduction with adenoviral NgBR. Therefore, these data demonstrated the significant role of C‐terminus of NgBR in stabilizing NPC2 and modulating cholesterol trafficking via binding to NPC2.^6^, ^27^

Interestingly, a report found that NgBR expression levels are decreased in fatty liver and can regulate hepatic lipogenesis in an NPC2‐independent way. To elucidate the underlying pathophysiological mechanisms, the NgBR liver‐specific knockout mice were generated and increased free fatty acids (FFA) and triglycerides (TG) levels were found in the animal models’ livers. Furthermore, the liver‐specific loss of NgBR led to increased expression of the liver X receptor alpha (LXRα)‐targeted lipogenic genes, which regulated by AMP‐activated kinase α (AMPKα) signalling pathway.^28^ In conclusion, it is definitely that NgBR can promote NPC2‐dependent cholesterol trafficking and NPC2‐independent metabolism of FFA and TG.

## 4 NgBR FACILITATES DOLICHOL SYNTHESIS AND PROTEIN N‐GLYCOSYLATION

*Cis*‐PTase is the first enzyme involved in the synthesis of dolichol which is an isoprenoid lipid, comprised of 15‐23 isoprene units^29^, ^30^ and essential for protein N‐glycosylation, O‐mannosylation and GPI anchor glycosylation.^7^, ^31^ Notably, previous studies have demonstrated that conserved C‐terminus of NgBR shares homology with cis‐PTase, which includes two yeast genes (*RER2* and *SRT1*), a human gene (*hCIT*/*DHDDS*) and bacterial undecaprenyl pyrophosphate synthase (*uppS*).^31^ Excitingly, the direct interaction exists between the C‐terminus of NgBR and hCIT, which yield a regulation effect of NgBR on dolichol synthesis. Moreover, the loss of NgBR causes a severe deficiency in cis‐PTase activity and dolichol synthesis, resulting in decreasing levels of dolichol‐linked oligosaccharides and protein N‐glycosylation. Interestingly, combined with the previous research results, the C‐terminus of NgBR likely exists two alternative topological conformations: one in the lumen of ER to interact with NPC2 and the other in the cytosol to bind hCIT.^7^

To date, several gene mutations have been reported to influence the dolichol biosynthesis pathway, such as mutations in *hCIT*/*DHDDS*,^32^, ^33^ *SRD5A3* ^34^, ^35^ and NgBR.^31^ Specifically, the NgBR‐R290H mutation, located in conserved C‐terminus of NgBR, was found in a family with congenital disorders of glycosylation (CDG), a genetic disease due to the defects in protein glycosylation. Analysis of the fibroblasts isolated from NgBR‐R290H patients revealed a decrease in the synthesis of dolichol and an increase in free cholesterol levels, consistent with the results found in fibroblasts that isolated from NgBR‐deficient mice. Additionally, the hyperglycosylated lysosomal associated membrane protein‐1 (LAMP‐1) and intercellular cell adhesion molecule‐1 (ICAM‐1) were found in patient fibroblasts, which reflects the loss function of NgBR in regulating protein N‐glycosylation. However, Nogo‐B/NgBR axis signal does not affect cholesterol trafficking or glycosylation.^31^ Similarly, another study demonstrates that NgBR plays a pivotal role in Nogo‐B‐independent angiogenesis by regulating the N‐linked glycosylation of EC proteins, such as VEGFR2, VE‐cadherin and CD31.^36^ In summary, conserved C‐terminus of NgBR functions in facilitating dolichol synthesis and protein N‐glycosylation.

## 5 NgBR PROMOTES VASCULAR REMODELLING AND ANGIOGENESIS

The formation and maturation of blood vessels is essential for embryogenesis, which implicated in the coordinated development of EC and VSMC.^37^

Notably, in vitro study shows that NgBR is necessary for Nogo‐B‐induced chemotaxis and morphogenesis of EC.^5^ To explore the Nogo‐B/NgBR function in vivo, Zhao et al found that knockdown of Nogo‐B or NgBR leads to the deficiency of intersomitic vessel (ISV) sprouting in zebrafish embryos. Additionally, knockdown of NgBR not only abrogated the Nogo‐B‐mediated migration of EC, but also attenuated vascular endothelial growth factor (VEGF)‐mediated Akt phosphorylation as well as VEGF‐mediated human umbilical vein endothelial cells (HUVECs) chemotaxis and morphogenesis. Furthermore, the activation of Akt or human NgBR can rescue above mentioned defects both in vitro and in vivo.^38^ To further demonstrate the regulatory mechanisms of NgBR on angiogenesis, Rana et al performed experiments in embryoid body culture systems; the results indicated that NgBR homozygous knockout mice and EC‐specific NgBR knockout mice are embryonically lethal with severe damage of vascular function. Moreover, in embryonic stem cells, the homozygous knockout of NgBR caused cell apoptosis while heterozygous knockout of NgBR disrupted the formation and branching of blood vessels without causing cell death. Besides, the significantly decreased NgBR expression levels are found in CD31 positive EC of human cerebral cavernous malformation (CCM) patient tissue sections.^39^ Taken together, the findings from these studies have greatly expanded our knowledge of NgBR in regulating EC migration and angiogenesis.

Persistent pulmonary hypertension of the newborn (PPHN) is a neonatal disease characterized by the impairment of pulmonary blood vessels.^40^ Previous reports found that increased reactive oxygen species (ROS) formation can destruct the angiogenesis of pulmonary artery endothelial cells (PAECs), which isolated from lungs of the intrauterine pulmonary hypertension (IPH) foetal lamb model.^41^, ^42^ Subsequently, Teng et al found decreased NgBR expression in IPH foetal lamb model, which led to the disruption of angiogenesis in PAECs. In contrast, overexpression of NgBR in IPH PAECs led to improved angiogenesis and increased levels of manganese superoxide dismutase (MnSOD) and GTP cyclohydrolase‐1 (GCH1) proteins.^43^ Nevertheless, the increased proliferation of pulmonary artery smooth muscle cells (PASMCs) was found in foetal lambs with chronic IPH.^44^ Further research found that decreased expression of NgBR contributes to the thickening of smooth muscle cell layer in PPNH.^45^ Likewise, the negative correlation between NgBR expression and VSMC proliferation was demonstrated by Yang et al using a rat model of pulmonary hypertension (HPH).^46^ In a recent published study, the increased expression of NgBR and decreased proliferation of VSMC were found in rats with diabetic erectile dysfunction (ED), whereas knockdown of NgBR can relieve ED.^47^ Thus, unlike the positive regulation of NgBR in promoting EC proliferation, NgBR can inhibit VSMC proliferation, and the possible mechanisms will be discussed below.

The essential role of VEGF‐dependent activation of phosphatidylinositol 3‐kinase (PI3K) and Akt in EC migration and survival have been confirmed,^48^ as well as in VEGF‐mediated angiogenesis both in vitro and in vivo.^49^ However, knockdown of NgBR in zebrafish results in much severe defects in ISV compared with Nogo‐B knockdown, which indicates the existence of other signalling pathways in addition to Nogo‐B/NgBR axis.^38^ To note, the activated myristoylated Akt (myrAkt) can rescue VEGF receptor inhibitor PTK787‐induced ISV deficiencies ^50^ as well as NgBR knockdown‐mediated ISV formation deficiencies.^38^ In vitro, the activated myrAkt can rescue the VEGF‐induced EC migration defects caused by NgBR knockdown.^38^ In turn, overexpression of NgBR can rescue defective angiogenesis by increasing the phosphorylation levels of Akt in IPH PAECs as well.^43^ In another independent study, knockdown of NgBR not only reduced EC migration by eliminating Akt phosphorylation, but also decreased the expression of both *CCM1* and *CCM2* genes, which associated with human CCM. Further in vitro and in vivo researches confirmed that the expression level of NgBR is positively correlated with CCM1 and CCM2 protein, which may be the pathological basis of CCM, but the specific pathogenesis needs further research.^39^ Therefore, NgBR can regulate EC migration and angiogenesis via VEGF‐dependent activation of Akt.

Endothelial nitro oxide (NO) synthase (eNOS) coupling is another signalling pathway involved in NgBR‐modulated angiogenesis. In a previous study, the eNOS coupling was found destructed in IPH PAECs.^41^ Surprisingly, overexpression of NgBR increased the phosphorylation of eNOS in IPH PAECs, whereas knockdown of NgBR led to decreased NO and increased ROS levels, thus demonstrated the relevance between NgBR‐modulated angiogenesis and eNOS coupling.^43^ Additionally, miR‐26a, a miRNA that binds to 3'‐UTR of NgBR, is predicted to be a key regulator in VEGF‐mediated angiogenesis of EC. The overexpression of miR‐26a in EC results in decreasing eNOS phosphorylation and NO level, which is important for VEGF‐mediated angiogenesis.^51^ These studies implied that NgBR is positively related to Nogo‐B and VEGF‐induced EC angiogenesis by modulating the phosphorylation of eNOS.

Specifically, in pathological conditions such as diabetic kidney disease, the interaction between NgBR and sNogo‐B plays an important role in vascular remodelling through dampening VEGF‐A signalling and reducing eNOS, Akt and GSK3β phosphorylation, which in some extent different from the physiological conditions.^19^

Reactive oxygen species formation can facilitate the growth and migration of VSMC by phosphorylating Akt and extracellular signal‐regulated kinase (ERK).^52^, ^53^, ^54^, ^55^ Importantly, Tadokoro et al found that decreased NgBR expression is associated with increased phosphorylation of ERK and ER stress, indicating the modulation effects of NgBR on ROS formation and remodelling of pulmonary arteries in hypertensive foetal lamb (HTFL) PASMCs.^45^ Moreover, knockdown of NgBR leads to the disruption of mitochondria‐associated membranes (MAM), an ultrastructure that transfers ER Ca^2+^ between mitochondria and ER. Additionally, decreased NgBR also results in increased pAkt‐induced phosphorylation of inositol 1,4,5‐trisphosphate receptor type 3 (IP_3_R3), decreased Ca^2+^ and increased HIF‐1α nuclear localization, which further promotes the proliferation of VSMC. By contrast, overexpression of NgBR can inhibit hypoxia‐induced VSMC proliferation via attenuating MAM‐regulated signal intensity.^46^ Finally, in the rats with diabetic ED, the expression levels of NgBR and ICAM‐1, as well as its correlated factors including steroid receptor coactivator (SRC) and proline‐rich tyrosine kinase 2 (PYK2) are increased, which provide a potential therapeutic option for diabetic ED via silencing NgBR or ICAM‐1.^47^ Thus, Akt/ERK phosphorylation or MAM‐mediated signalling pathway or recently identified ICAM‐1 expression can regulate VSMC migration and proliferation.

Briefly speaking, NgBR is critical for vascular remodelling and angiogenesis both in physiological and pathological conditions, which regulated by a variety of signalling factors, such as Akt, eNOS and ERK. And new signalling factors are about to be disclosed. Certainly, these factors will provide potential targets for therapy in patients.

## 6 THE REGULATION OF NgBR IN TUMORIGENESIS

In the past few years, there have been plenty of studies focusing on the relationship between NgBR and tumorigenesis. And the correlation has been confirmed in some neoplastic diseases, such as breast invasive ductal carcinoma (IDC),^8^, ^9^, ^10^ non‐small cell carcinomas (NSCLC),^56^ malignant melanoma (MM)^11^ and human hepatocellular carcinoma (HCC)^57^, ^58^; however, the exact pathological mechanism remains to be clarified.

Breast cancer is the most common malignant disease in the female population with the highest incidence and mortality.^59^ Using immunohistochemistry approach, Wang et al first investigated the expression of NgBR in breast tumour tissues and normal breast tissues, and the results showed that NgBR protein is highly expressed in estrogen receptor alpha (ERα)‐positive/Her2‐negative breast tumour cells. Additionally, the gene expression of survivin, a well‐known apoptosis inhibitor, was found positively related to the expression of NgBR in ERα‐positive/Her2‐negative IDC cells, which was further confirmed by real‐time PCR. Furthermore, in vitro studies showed that estradiol induces the survivin expression exclusively in ERα‐positive breast tumour cells, whereas knockdown of NgBR with small interfering RNA (siRNA) abrogated the survivin expression. Taken together, NgBR is highly expressed in ERα‐positive breast tumour cells and positively correlated with the expression of survivin.^8^ To identify the aforementioned results, Pula et al examined NgBR expression between IDC patient breast tissues and non‐malignant breast tissues (NMBT), and surprisingly, they found the NgBR immunoreactivity was negatively correlated with the malignancy grades of IDC and expression levels of Ki‐67 antigen.^9^ Another research further demonstrated that knockdown of NgBR can block epithelial‐mesenchymal transition (EMT),^10^ which plays a pivotal role in the metastasis of breast cancers.^60^, ^61^ In summary, NgBR can promote the progression and distant metastasis of ERα‐positive IDC.

On the contrary, Pula et al did not observe any differences in the levels of NgBR mRNA between non‐malignant tissue (NMLT) and NSCLC. To note, the expression of NgBR mRNA was negatively associated with the tumour size, lymph node involvement and advancement stage. Additionally, the low NgBR expression indicates the poor prognosis; thus, these results provide evidence that NgBR may contribute to the progression of NSCLC.^56^ In MM, the expression of Nogo‐B and NgBR were correlated negatively with the depth of primary MM tumour invasion, melanoma cell migration and invasiveness, indicating that NgBR may function as an onco‐suppressor gene.^11^ Another study demonstrated that increased expression of NgBR lead to increased chemoresistance of Bel7402/5FU cells, and the poor prognosis was found correlated with the higher NgBR expression in HCC patients.^57^ Taken together, NgBR may serve as an oncogene for IDC and HCC, but as an onco‐suppressor gene for NSCLC and MM.

Although several lines of evidence have suggested that the disturbance of NgBR expression level is correlated with tumorigenesis, the underlying molecular mechanism remains elusive. As we all know, Ras is an oncogene that can cause tumorigenesis and drug resistance through the phosphatidylinositol‐3‐OH kinase (PI3K)/Akt and Raf1/ERK pathways.^62^, ^63^, ^64^, ^65^, ^66^ Phosphorylated and activated Akt can regulate protein synthesis, cell proliferation, survival and angiogenesis by phosphorylating its downstream signal effectors.^67^, ^68^ A previous study found that the hydrophobic cytoplasmic domain of NgBR binds farnesylated Ras and further promotes the plasma membrane accumulation of Ras, which is a critical step for the activation of the epidermal growth factor (EGF)‐stimulated Ras pathway in human breast cancer cells.^69^ Furthermore, Dong et al found that knockdown of NgBR lead to an overt loss of phosphorylated Akt in human HCC cells (HepG2 and SMMC‐7721) compared with the normal liver cells, but overexpression of NgBR can rescue the impaired phosphorylated Akt levels in human HCC cells.^58^ Moreover, Akt mediates phosphorylation of MDM2 and then promotes the ubiquitination and degradation of p53.^70^, ^71^, ^72^, ^73^ Surprisingly, the PI‐3K/Akt/MDM2‐mediated degradation of p53 protein is enhanced by NgBR expression and thus exerts the chemoresistance to Bel/5FU.^57^ Similarly, the increased expression of NgBR is also found in tamoxifen and paclitaxel‐resistant ERα‐positive breast cancer cell lines. And the increased expression of NgBR promotes the EGF‐stimulated Ras activation and Akt/ERK‐mediated MDM2 phosphorylation, which leads to decreased p53 and increased survivin expression.^74^, ^75^

Notably, Raf‐1/ERK pathway is also involved in tumorigenesis besides PI3K/Akt signalling pathway. Similar to what found in breast cancer cells, a recent study showed that NgBR can active Ras by promoting its plasma membrane localization in NSCLC cells. Further experiments found that knockdown of NgBR can destroy the localization and activation of Ras, whereas NgBR overexpression promotes the MEK/ERK/Snail 1‐mediated Ras signalling pathway. However, both knockdown and overexpression of NgBR affected Akt activation. Taken together, these results indicate that NgBR can promote EMT in NSCLC cells via Ras/Raf‐1/MEK/ERK/Snail 1 pathway.^76^ Nevertheless, the role of NgBR in MM has not been elucidated.

In summary, NgBR may exert completely opposite effects on the progression of different tumours, either promote or inhibit tumorigenesis. However, the molecular mechanisms of NgBR in tumorigenesis are very complicated. Apart from Ras‐related PI3K/Akt and Raf‐1/ERK pathways, other signalling pathways may also affect tumorigenesis.

## 7 THE REGULATION OF NgBR IN NERVOUS SYSTEM

As for the central nervous system (CNS), researchers found NgBR highly expressed in the soma and axonal processes of sensory neurons. Specifically, the interaction between Schwann cells expressed Nogo‐B and neuronal NgBR was demonstrated. More importantly, the interaction can further modulate axonal branching other than activating axonal long‐distance growth; however, the effect is restricted to immature, undifferentiated Schwann cells.^77^ Considering the previous study found that PI3K/Akt signalling participates in promoting neuronal survival, axonal growth and axonal branching, whereas ERK/MAPK signalling only promoting axonal extension,^78^ this glia‐neuron crosstalk implies a novel regulation mechanism of Nogo‐B/NgBR axis signal for axonal branching via activating PI3K/Akt signalling pathway.

*NUS1* encodes a precursor of NgBR. Interestingly, recent studies found that mutations in *NUS1* cause not only neurodevelopmental disorders but also neurodegenerative disease.^12^, ^79^ In a clinical trial, six patients had no consanguinity with non‐recurrent deletions in 6q22.1 region including *NUS1* and established that the loss of this region results in a neurodevelopmental disorder with clinical features such as epilepsy, cognitive deficits, language delay and variable kinds of tremors.^79^ As mentioned above, *NUS1* as well as its orthologue NgBR can facilitate protein N‐glycosylation, so that its deficiency may cause CDG.^7^, ^31^ According to the research, CDG presents as epileptic encephalopathy with migrating seizures in infancy.^80^ Therefore, it is tempting to speculate that the occurrence of paediatric epilepsy may be due to defects in glycosylation caused by NgBR dysfunction. The emergence and development of the next‐generation sequencing (NGS) technology has derived the whole exome sequencing (WES), the whole genome exon sequencing (WES) and other technologies. The NGS is a key to the research of nervous system diseases and plays a great role in the research of pathogeny, clinical diagnosis, prognosis and treatment.^81^, ^82^, ^83^, ^84^ Lately, Guo et al analysed 39 core families of early‐onset Parkinson's disease (EOPD) through the WES technology to select out 12 new candidate genes. Subsequently, a two‐stage verification was conducted to identify that PD patients carry more rare mutations in *NUS1*. This makes this gene particularly suspicious. Since then, the researchers observed PD‐related phenotypes such as decreased exercise, loss of dopaminergic neurons and decreased dopamine transmitters in knocking down the orthologous gene of *NUS1* in *Drosophila*. All of these findings suggest that *NUS1* may play a role in the development of PD.^12^ At present, our work is still concerned about the impact of *NUS1* on the development of PD. Furthermore, we systematically evaluated common and low‐frequency variants in a discovery sample set and then successfully replicated findings in another large case‐control sample to result in that common and low‐frequency variants in *NUS1* have an effect on the pathogenesis of PD and may influence EOPD onset age (unpublished). Overall, from the current results, NgBR is not only involved in the branching of sensory neuron axons, but also related to several neurological diseases, such as paediatric epilepsy and PD. Identification of *NUS1* as PD‐causing gene will further expand understanding the molecular pathogenesis of disease. However, the regulation of NgBR in the nervous system still remains elusive, which is worth exploring further in the future.

## 8 CONCLUSIONS

**FIGURE 2:**
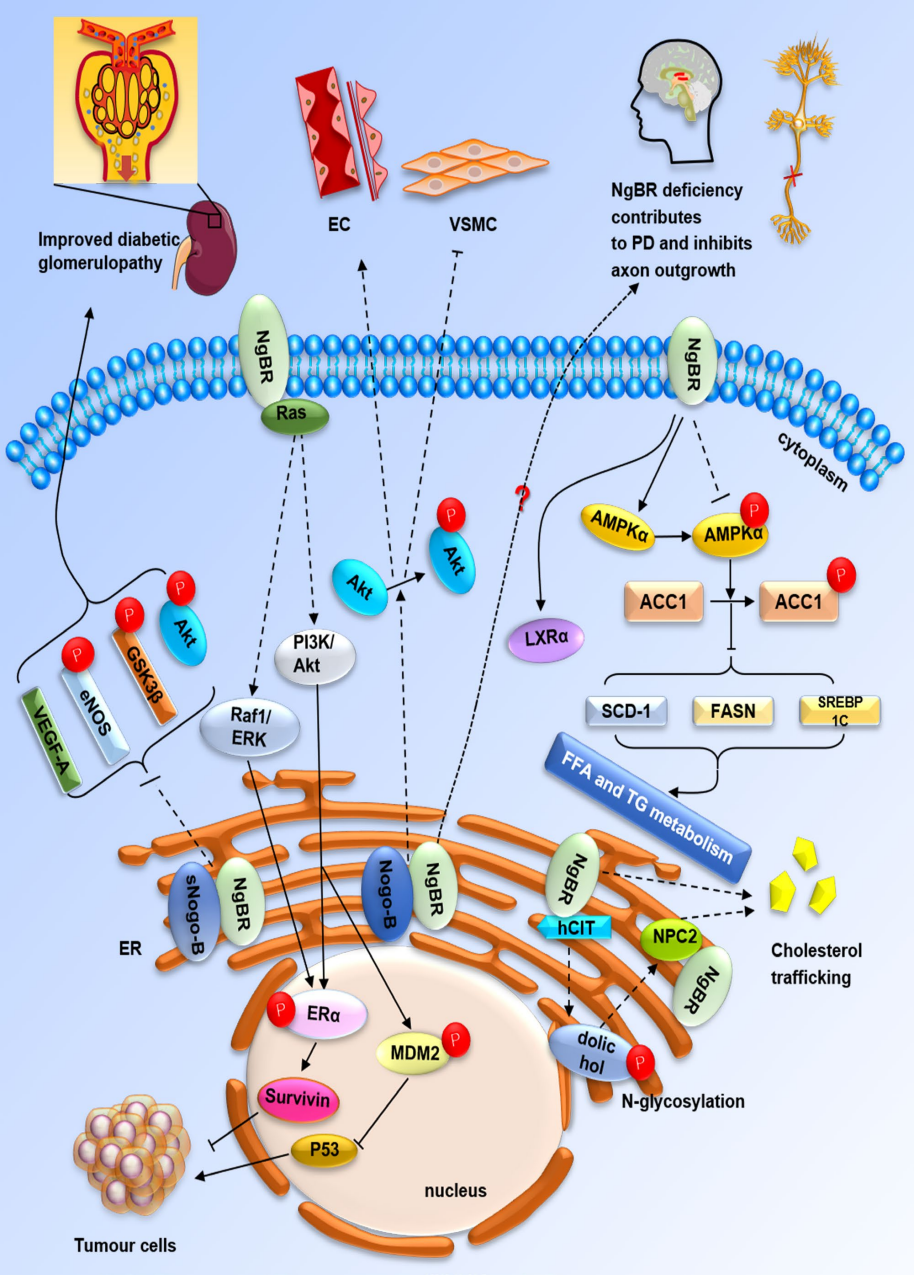
The functions of NgBR include regulating cholesterol trafficking via directly binding to NPC2, hepatic FFA and TG metabolism through inhibiting LXRα transcription in an AMPKα‐dependent pathway. In addition, NgBR facilitates dolichol synthesis and further induces protein N‐glycosylation when binds to hCIT and Nogo‐B/NgBR axis promotes vascular remodelling and angiogenesis via VEGF‐mediated Akt phosphorylation. Particularly, sNogo‐B/NgBR lead to improved diabetic glomerulopathy by dampening VEGF‐A signalling and reducing eNOS, Akt and GSK3β phosphorylation. Moreover, NgBR regulates tumorigenesis via Ras‐related PI‐3K/Akt and Raf‐1/ERK pathways. Finally, NgBR deficiency contributes to PD and inhibits axon outgrowth. Endoplasmic reticulum (ER); estrogen receptor alpha (ERα); free fatty acid (FFA); Triglyceride (TG); Vascular smooth muscle cells (VSMC)

Structural‐functional correlation analysis shows that C‐terminus of NgBR can modulate NPC2‐dependent cholesterol trafficking and NPC2‐independent lipid metabolism, as well as facilitate dolichol synthesis and protein N‐glycosylation, whereas AmNgBR can promote vascular remodelling and angiogenesis. Besides, a hydrophobic cytoplasmic domain of NgBR can bind farnesylated Ras and further involved in tumorigenesis. The integral characteristics of NgBR mentioned above are presented in Figure 2. In conclusion, the expression of NgBR is essential for the maintenance of normal physiological functions, such as regulating cholesterol trafficking, lipid metabolism, dolichol synthesis, protein N‐glycosylation, vascular remodelling, angiogenesis and tumorigenesis. Changes in NgBR expression levels may lead to a group of human diseases, such as NPC, fatty liver, CDG, PPHN, IDC, MM, NSCLC and nervous system diseases.

Despite multiple features of NgBR have been reported, it is just the tip of the iceberg. In the future, it is worthy further study to lucubrate the structural characteristics and functions of NgBR. Meanwhile, we should expand our perspectives and find out more possible regulatory mechanisms, thus interpreting the unresolved issues. Last, these findings may provide us strong implications for the development of therapeutic strategies of NgBR‐related diseases.

## CONFLICT OF INTERESTS

The authors declare no competing interests.

## AUTHOR CONTRIBUTIONS

Rui Zhang and Beisha Tang performed most of the writing, original draft preparation and visualization; Beisha Tang conducted data curation and writing revising; Beisha Tang and Jifeng Guo participated in final interpretation. All the authors discussed the review and commented on the manuscript.
